# Development of a Novel Predictive-Prognostic Scoring Index for Immune Checkpoint Inhibitors in Advanced Non-small Cell Lung Cancer

**DOI:** 10.7759/cureus.33234

**Published:** 2023-01-01

**Authors:** Omer Diker, Polat Olgun, Ugurcan Balyemez, Sinem Sigit Ikiz

**Affiliations:** 1 Medical Oncology, Near East University Hospital, Nicosia, CYP; 2 Radiology, Near East University Hospital, Nicosia, CYP

**Keywords:** cel score, prognostic biomarker, risk scoring system, ecog ps 2, advanced non-small cell lung cancer

## Abstract

Background: Immune checkpoint inhibitors (ICIs) have become the standard of care for the treatment of patients with driver mutation absent advanced non-small cell lung cancer (NSCLC). The present study aimed to develop a reliable, reproducible, and practical scoring system to prognosticate and predict response to ICI response in patients with advanced NSCLC.

Patients and methods: All patients who were diagnosed as having unresectable/advanced stage NSCLC and were treated with at least one cycle of ICIs at the Medical Oncology Departments of Dr. Burhan Nalbantoğlu State Hospital (Nicosia, Cyprus) and Near East University Hospital (Nicosia, Cyprus) were included in the study.﻿ The association between variables and OS was evaluated using a Cox ﻿proportional hazards regression model. Variables with a P-value less than 0.05 in the univariate analysis were included in the multivariate model. A prognostic scoring system was developed. ﻿ Survival estimates were calculated using the Kaplan-Meier method. The value of the Concordance index (C-index) and the area under the curve ﻿(AUC) was used to evaluate the discriminative ability of scoring systems.

Results: One hundred fifty consecutive patients with unresectable/metastatic NSCLC who received PD-1 inhibitors ﻿between March 2017 and November 2022 were included. In the multivariate Cox regression model, serum lactate dehydrogenase (LDH), C-reactive protein (CRP) levels, and Eastern Cooperative Oncology Group Performance Status (ECOG PS) were significantly associated with OS. We generated a new score using CRP ³1.0 mg/dL, ECOG PS ³2, and LDH level >ULN. Relative weight was based on the HRs of multivariate analyses (CRP ³1.0 mg/dL 2 points, ECOG PS ³2 2.5 points, and LDH level >ULN 1.5 points). The cohort was divided into three risk groups based on the sum of factors present: 0-2.5 (good risk), 3.5-4.5 (intermediate risk), or 6 (poor risk). The median OS was 18.9, 7.4, and 2.9 months for good, intermediate, and poor risk categories, respectively (log-rank test, p<0.001). The Harrell C-index of CEL to predict OS and PFS was 0.73 and 0.69, respectively, indicating significant predictability. The AUC of the scoring index for predicting the responses was 0.765 (95% CI: 0.685-0.845).

Conclusion: The CEL score is a promising prognostic and predictive index consisting of serum CRP levels (C), ECOG PS (E), and serum LDH levels (L). This represents another step forward in the treatment of patients with advanced NSCLC.

## Introduction

Immune checkpoint inhibitors (ICIs) have become the standard of care for the treatment of patients with driver mutation absent advanced non-small cell lung cancer (NSCLC). Although ICIs are a revolutionary treatment option, more than half of all patients with advanced NSCLC do not respond to ICIs [1,2]. ﻿Treatment with ICIs also ﻿leads to a serious financial burden. Patients with driver mutation absent advanced NSCLC are stratified according to their programmed death-ligand 1 (PD-L1) levels for the initial choice of treatment; if the PD-L1 level is higher than 50% treatment is started with pembrolizumab, if lower than 50%, a chemotherapy immunotherapy combination is preferred.

 There are ongoing controversies about whether PD-L1 is optimal, such as the emergence of responses in PD-L1-negative patients, heterogeneity of PD-L1 expression in serial tumour sections, and changes in expression with treatments. Moreover, there are no reliable and practical biomarkers for platinum-refractory patients with NSCLC [3-5].

Although immunotherapy has remarkable heterogeneity regarding its objective response rate and survival, we and others have tried to optimise patient benefit by using various parameters, such as clinicopathologic factors [6-9], laboratory (neutrophil-to-lymphocyte ratio [NLR] [10], platelet-to-lymphocyte ratio [10], lactate dehydrogenase [LDH] [6,11,12], serum C-reactive protein [CRP] [6,13], and tumour markers [14]), and body composition parameters [15]. Although these studies showed an association with survival and responses, they did not provide complete quantitative prediction and prognostication. Some composite scoring systems such as the Royal Marsden Hospital score (RMH), ﻿Gustave Roussy Immune Score (GRIm-Score), Lung Immune Prognostic Index (LIPI), and modified Glasgow Prognostic Score (mGPS) were developed and validated in several cohorts of patients with advanced NSCLC but remain underused in everyday clinical practice [11,12,16-18]. In addition, some studies have shown that they sometimes perform poorly in risk stratification [19]. ﻿Scoring systems guide treatment selection in patients with hepatocellular and metastatic renal cell carcinoma [20,21]. This has inspired us to develop a scoring index that can guide the ICI treatment of NSCLC.

In this context, the present study aimed to develop a reliable, reproducible, and practical scoring system to prognosticate and predict response to ICI response in patients with advanced NSCLC. Finally, we compared the performance of our scoring index with the RMH, GRIm-Score, LIPI, and mGPS.

## Materials and methods

Study population

﻿All patients who were diagnosed as having unresectable/advanced stage NSCLC and were treated with at least one cycle of ICIs at the Medical Oncology Departments of Dr. Burhan Nalbantoğlu State Hospital (Nicosia, Cyprus) and Near East University Hospital (Nicosia, Cyprus) were included in the study.﻿

Data acquisition

Patient characteristics, laboratory parameters, and treatment information were obtained from patient files, chemotherapy unit files, and the electronic record system of each hospital. The following patient demographics were recorded for analysis: sex; histologic subtype of NSCLC; age; Eastern Cooperative Oncology Group Performance Status (ECOG PS); smoking history; molecular alteration profile when available; PD-L1 status (Dako; Carpinteria, CA, USA) when available; sites of metastasis; date of progression, death or last follow-up; immune-related adverse events (irAEs); platelet count, white blood cell (WBC) count, absolute neutrophil count (ANC), absolute lymphocyte count (ALC), serum albumin, lactate dehydrogenase (LDH), and C-reactive protein (CRP) levels (defined as the most recent drawn sample within two weeks before the initiation of ICI treatment).

The RMH, GRIm-Score, LIPI, and mGPS were also calculated for each patient as a composite scoring system, based on the clinical and laboratory values [11,16,19,22]. The RMH score is calculated based on ﻿LDH levels (normal: 0; >upper limit of normal [ULN]: 1), the number of metastasis (<3: 0; ³3: 1), and albumin levels (³3.5 g/dL: 0; <3.5 g/dL: 1). Patients who had an RMH score of 2-3 were categorised as high risk, and scores 0-1 were categorised as low risk [19]. ﻿The GRIm-Score was calculated based on the NLR, LDH, and serum albumin. Patients were assigned 1 point for each of the following: NLR >6, LDH >ULN, or albumin <3.5 g/dL, for a total of 3 points. GRIm-Scores ³2 were considered high risk [16]. ﻿The LIPI was calculated based on dNLR (>3: 1, £3: 0) and LDH (>ULN: 1, £ULN), and patients were stratified into three groups (good: 0; intermediate: 1; poor: 2) [11]. ﻿The mGPS was calculated based on serum CRP and albumin levels. Patients with serum CRP <1.0 mg/dL with or without hypoalbuminemia were grouped as mGPS:0; elevated CRP (³1.0 mg/dL) and hypoalbuminemia (<3.5 g/dL) were assigned as mGPS:2. Patients with only elevated CRP levels scored mGPS:1 [22].

Tumour evaluation

Tumour response was evaluated through computed tomography or fluorodeoxyglucose positron emission tomography-CT every three months according to response evaluation criteria in solid tumours criteria (RECIST) V1.1 [23].

Statistical analysis and scoring system development

Progression-free survival (PFS) was calculated as the number of months between the first day of ICI treatment and disease progression or death. Overall survival (OS) was calculated as the number of months between the first day of ICI treatment and death. The sum of complete (CR) and partial response (PR) percentages are defined as the objective response rate (ORR).

The association between variables and OS were evaluated using a Cox ﻿proportional hazards regression model. Variables with a P-value less than 0.05 in the univariate analysis were included in the multivariate model.

﻿Survival estimates were calculated using the Kaplan-Meier method. The value of the Concordance index (C-index) and the area under the curve ﻿(AUC) was used to evaluate the discriminative ability of scoring systems [24]. ﻿All statistical analyses were performed using the SPSS version 22 software (IBM Corp., Chicago, IL) and Stata/IC version 14.2 for Windows (Stata Corp LLC, ﻿College Station, TX).

Ethics

Ethical approval was obtained from the institutional board. A written informed consent waiver was granted because of the retrospective nature of the study. ﻿All study procedures were performed in accordance with the 1964 Declaration of Helsinki and its later amendments.

## Results

Patient characteristics

One hundred fifty consecutive patients with unresectable/metastatic NSCLC who received PD-1 inhibitors ﻿between March 2017 and November 2022 were included. The median age of the patients was 68.0 (range, 35.0-88.0) years. ﻿The majority of the patients were male (n=131, 87.3%) and former or current smokers (n=142, 94.7%). Eighty-five (56.7%) patients had an ECOG PS ³2, 36.0% of the patients were diagnosed as having squamous cell carcinoma, and 20.0% of patients had liver metastasis. ﻿The other clinical characteristics and laboratory parameters are shown in Table 1.

**Table 1:** Baseline patient and tumor characteristics

Age at the start (years)	
Median	68.0
Range	35-88
Gender-n (%)	
Male	131 (87.3)
Female	19 (12.7)
Smoking status-n (%)	
Current or former smoker	142 (94.7)
Never smoked	8 (5.3)
Histology-no (%)	
Non-squamous	96 (64.0)
Squamous cell carcinoma	54 (36.0)
PD-L1-n (%)	
Negative	42 (28.0)
1-49%	12 (8.0)
³50%	13 (8.7)
Unknown	83 (55.3)
Driver mutations-n (%)	
EGFR mutation	1 (0.7)
ALK translocation	1 (0.7)
Not assessed	54 (36.0)
EGFR-ALK wild type	94 (62.6)
ECOG performance status-n (%)	
0-1	65 (43.3)
2-4	85 (56.7)
Number of metastases-n (%)	
1-2	122 (81.3)
³3	28 (18.7)
CNS metastasis-n (%)	
Yes	19 (12.7)
No	131 (87.3)
Liver metastasis-n (%)	
Yes	30 (20.0)
No	120 (80.0)
Bone metastasis-n (%)	
Yes	62 (41.3)
No	88 (58.7)
Malignant pleural effusion-n (%)	
Yes	35 (23.3)
No	115 (76.7)
Adrenal gland metastasis-n (%)	
Yes	28 (18.7)
No	122 (81.3)
White blood cells-n (%)	
>10,000/mm^3^	52 (34.7)
£10,000/mm^3^	98 (65.3)
Neutrophil-to-lymphocyte ratio-n (%)	
³6	46 (30.7)
<6	104 (69.3)
Derived neutrophil-to-lymphocyte ratio-n (%)	
>3	45 (30.0)
£3	105 (70.0)
Platelets-n (%)	
>400,000/mm3	36 (24.0)
<400,000/mm3	114 (76.0)

Treatment and survival

In the entire patient population, ﻿46.0% of the patients were treatment-naive and 54.0% were platinum-pretreated. The median duration of follow-up was 33.0 (range, 0.03-62.90) months. At the time of the database lock (November 28, 2022), 22.0% of patients continued ICI treatment. ﻿Treatment characteristics are presented in Table 2. One hundred two (68.0%) patients died. The median PFS and OS was 5.2 months (95% confidence interval (CI): 3.7-6.8) and 10.8 months (95% CI: 7.2-14.3), respectively, and the ORR was 46.2%.

**Table 2:** Treatment characteristics of the patients.

Variables	Patients (N=150)
First-line immune checkpoint inhibitors-n (%)	69 (46.0)
Single-agent pembrolizumab	13 (8.6)
Pembrolizumab plus chemotherapy	45 (30.0)
Nivolumab plus ipilimumab	9 (6.0)
Nivolumab plus chemotherapy	2 (1.4)
³ 2nd-line immune checkpoint inhibitors-n (%)	81 (54.0)
Single-agent pembrolizumab	5 (3.3)
Single agent nivolumab	75 (50.0)
Nivolumab plus ipilimumab	1 (0.7)
Reasons of discontinuation for ICIs-n (%)	
Progressive disease or death	105 (70.0)
IRAEs	1 (0.7)
No evidence of disease >1 year with ICI	11 (7.3)

﻿Univariate analysis and multivariate analysis

Eleven prognostic factors were identified in the univariate analysis. Liver metastasis (hazard ratio [HR] 2.10, 95% CI: 1.33-3.31), bone metastasis (HR 1.56, 95% CI: 1.05-2.31), brain metastasis (HR 1.98, 95% CI: 1.14-3.45), number of metastases (HR 2.02, 95% CI: 1.24-3.28), WBC >10,000/mm3 (HR 2.31, 95% CI: 1.54-3.46), dNLR>3 (HR 1.68, 95% CI: 1.10-2.55), serum albumin <3.5 g/dL (HR 1.70, 95% CI: 1.08-2.67), LDH >ULN (HR 2.50, 95% CI: 1.66-3.76), CRP ³1.0 mg/dL (HR 2.17, 95% CI: 1.40-3.37), and ECOG PS ³2 (HR 2.60, 95% CI: 1.72-3.95) were associated with worse OS outcomes. ﻿Patients with unknown PD-L1 levels (HR 0.57, 95% CI: 0.36-0.91) showed better OS. In the multivariate Cox regression model, LDH >ULN (HR 1.75, 95% CI: 1.12-2.75), CRP ³ 1.0 mg/dL (HR 1.96, 95% CI: 1.20-3.19), and ECOG PS ³2 (HR 2.60, 95% CI: 1.61-4.19) remained statistically significant. Details of the univariate and multivariate analyses are shown in Table 3.

**Table 3:** Prognostic factors for overall survival (univariate analysis and multivariate analysis). HR: Hazard ratio, CI: confidence interval, NLR: neutrophil to lymphocyte ratio, LDH: lactate dehydrogenase, ULN: upper limit normal, ECOG PS: Eastern Cooperative Oncology Group Performance Status, CRP: C-reactive protein, TPS: tumour proportion score, dNLR: derived neutrophil to lymphocyte ratio, WBC: White blood cells

﻿Characteristic		﻿Univariate analysis			﻿Multivariate analysis	
	HR	﻿95% CI	P value	HR	﻿95% CI	P value
NLR>6	1.43	0.94-2.17	0.089			
LDH >ULN	2.50	1.66-3.76	<0.0001	1.75	1.12-2.75	0.014
Presence of liver metastasis	2.10	1.33-3.31	0.001	1.65	0.95-2.89	0.074
Presence of bone metastasis	1.56	1.05-2.31	0.027	1.12	0.69-1.83	0.623
Malignant pleural effusion	1.35	0.85-2.15	0.198			
Presence of brain metastasis	1.98	1.14-3.45	0.015	1.79	0.93-3.43	0.078
Presence of adrenal gland metastasis	1.16	0.72-1.86	0.530			
﻿Number of metastases ³3	2.02	1.24-3.28	0.004	1.38	0.67-2.80	0.373
﻿Albumin <3.5 g/dL	1.70	1.08-2.67	0.020	1.70	1.08-2.67	0.887
ECOG PS ³2	2.60	1.72-3.95	<0.0001	2.60	1.61-4.19	<0.0001
CRP ³1 mg/dL	2.17	1.40-3.37	0.001	1.96	1.20-3.19	0.006
Stage III vs IV	0.65	0.34-1.26	0.211			
﻿PD-L1 TPS% ﻿<1% (reference) 1%-49% ³50% Unknown	1 0.79 0.68 0.57	0.37-1.67 0.33-1.39 0.36-0.91	0.130 0.544 0.296 0.019	1 1.07 0.88 0.64	0.49-2.33 0.42-1.86 0.39-1.06	0.300 0.855 0.813 0.086
dNLR>3	1.68	1.10-2.55	0.014	1.12	0.69-1.80	0.633
Squamous histology	0.68	0.45-1.03	0.073			
Female gender	0.72	0.40-1.30	0.286			
Smoking status Never vs Smoker	0.82	0.36-1.88	0.651			
﻿WBC>10,000/mm^3^	2.31	1.54-3.46	<0.0001	1.51	0.93-2.46	0.093
﻿Platelet>400,000/mm3	1.43	0.91-2.24	0.117			

Construction and evaluation of a novel scoring system

**Figure 1:**
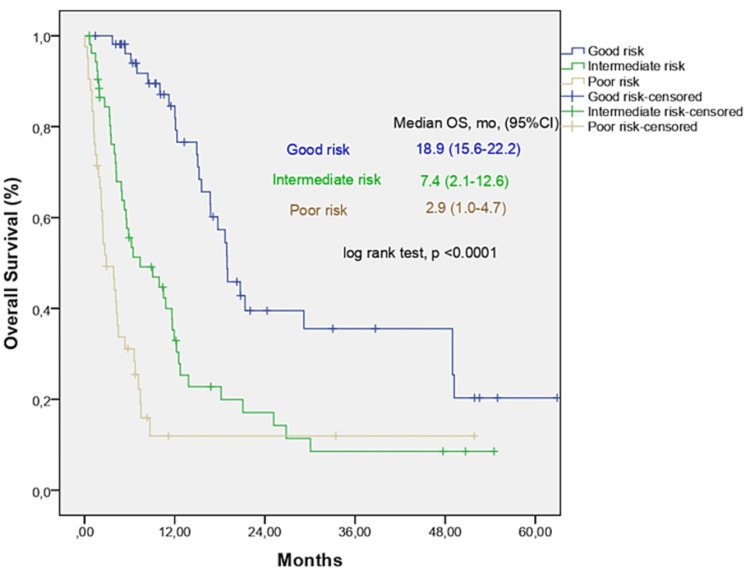
Overall survival (OS) analysis according to CEL score.

**Figure 2:**
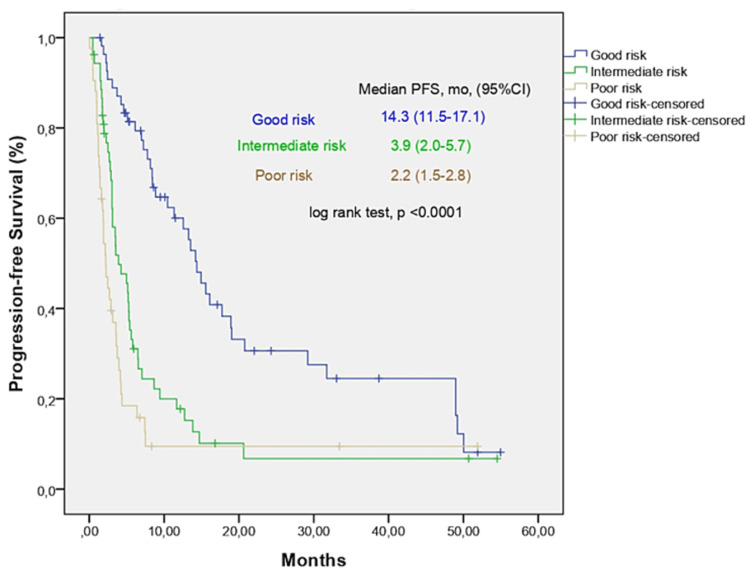
Progression-free survival (PFS) analysis according to CEL score.

We generated a new score using CRP ³1.0 mg/dL, ECOG PS ³2, and LDH level >ULN. Relative weight was based on the HRs of the multivariate analysis (CRP ³1.0 mg/dL 2 points, ECOG PS ³2 2.5 points, and LDH level >ULN 1.5 points). Twenty patients (13.3%) had 0 risk factors, and 28.0% had 3 risk factors. The cohort was divided into three risk groups based on the sum of factors present: 0-2.5 (good risk), 3.5-4.5 (intermediate risk), or 6 (poor risk). Median OS was 18.9, 7.4, and 2.9 months for the good, intermediate, and poor risk categories, respectively (log-rank test, p<0.001; Figure 1). The median PFS was 14.3, 3.9, and 2.2 months for the good, intermediate, and poor risk categories, respectively (log-rank test, p<0.001; Figure 2). The ORR was 77.8% in the good risk group, which was significantly higher than that in the intermediate (32.7%) and poor risk (20.0%) groups (p<0.001).

﻿Comparison of the new scoring index (CEL) with RMH, GRIm, mGPS and LIPI scores

The patient distribution for each scoring index is presented in Table 4. We compared the ability of our scoring index with the RMH, GRIm, mGPS, and LIPI. The Harrell C-index of CEL to predict OS and PFS was 0.73 and 0.69, respectively, indicating significant predictability. The AUC of the scoring index for predicting the responses was 0.765 (95% CI: 0.685-0.845). We performed a subgroup analysis for treatment-naive and platinum-pretreated patients to calculate the performance of our scoring index. The CEL scoring index showed significant discriminative ability regardless of the treatment line. The Harrell C-index of CEL to predict OS and PFS for treatment-naive patients was 0.76 and 0.71, respectively. The AUC was 0.817 (95% CI: 0.708-0.925). The Harrell C-index of CEL to predict OS and PFS for platinum-pretreated patients was 0.71 and 0.68, respectively. The AUC was 0.720 (95% CI: 0.604-0.835). We also calculated the Harrell C-index and AUC for the other scoring systems, and CEL had better discriminative and predictive abilities than the other scoring systems (Table 5).

**Table 4:** Patients distribution for each scoring index. mGPS: modified Glasgow Prognostic Score, LIPI: Lung immune prognostic index, RMH: Royal Marsden Hospital Score, GRIm: Gustave Roussy Immune Score, CEL: C-reactive protein, Eastern Cooperative Oncology Group Performance Status, Lactate dehydrogenase

Scoring Systems	Patients (N=150)
mGPS-n (%)	
0	45 (30.0)
1	74 (49.3)
2	31 (20.7)
LIPI-n (%)	
Good	60 (40.0)
Intermediate	66 (44.0)
Poor	24 (16.0)
RMH-n (%)	
Low risk	114 (76.0)
High risk	36 (24.0)
GRIm-n (%)	
Low risk	105 (70.0)
High risk	45 (30.0)
CEL-n (%)	
Good	55 (36.7)
Intermediate	53 (35.3)
Poor	42 (28.0)

**Table 5:** Distribution of objective response rate, progression-free survival, overall survival, and the predictive and discriminative ability for each scoring system. mGPS: modified Glasgow Prognostic Score, LIPI: Lung immune prognostic index, RMH: Royal Marsden Hospital Score, GRIm: Gustave Roussy Immune Score, CEL: C-reactive protein, Eastern Cooperative Oncology Group Performance Status, Lactate dehydrogenase, ORR: Objective response rate, OR: odds ratio, CI: confidence interval, ROC: Receiver operating curve analysis, AUC: area under curve, mPFS: median progression-free survival, mo: months, HR: hazard ratio, C index: Harrell’s C index (concordance index), mOS: median overall survival

Scoring Systems	ORR (%)	OR (95% CI)	p-value	ROC AUC (95% CI)	p-value	mPFS (mo) (95% CI)	HR (95% CI)	p-value	C index-PFS	mOS (mo) (95% CI)	HR (95% CI)	p-value	C index-OS
mGPS				0.644 (0.55-0.73)	0.003				0.62				0.64
0	64.4	4.75 (1.72-13.16)	0.003			13.5 (6.6-20.4)	1 (reference)	0.009		17.7 (13.3-22.0)	1 (reference)	0.004	
1	42.0	1.90 (0.74-4.89)	0.182			4.3 (3.3-5.3)	1.83 (1.20-2.81)	0.005		10.8 (6.1-15.4)	1.83 (1.15-2.92)	0.011	
2	27.6	1 (reference)	0.007			2.4 (1.3-3.5)	1.94 (1.14-3.31)	0.014		2.9 (1.4-4.3)	2.43 (1.40-4.23)	0.002	
LIPI				0.624 (0.53-0.71)	0.011				0.63				0.66
Good	60.7	3.31 (1.16-9.41)	0.025			13.5 (7.0-20.0)	1 (reference)	<0.001		17.7 (13.0-22.3)	1 (reference)	<0.001	
Intermediate	38.5	1.33 (0.48-3.74)	0.577			3.7 (2.7-4.7)	1.93 (1.28-2.90)	0.002		7.4 (4.5-10.2)	2.09 (1.34-3.26)	0.001	
Poor	31.8	1 (reference)	0.019			2.2 (0.4-3.9)	3.15 (1.84-5.38)	<0.001		2.9 (0.0-5.9)	3.60 (2.03-6.39)	<0.001	
RMH				0.644 (0.55-0.73)	0.003				0.62				0.64
Low risk	55.5	6.97 (2.50-19.39)	<0.001			7.7 (5.7-9.7)	1 (reference)			13.8 (10.7-16.9)	1 (reference)		
High risk	15.2	1 (reference)				2.2 (1.3-3.0)	4.31 (2.72-6.81)	<0.001		3.5 (1.6-5.4)	5.08 (3.20-8.05)	<0.001	
GRIm				0.611 (0.51-0.70)	0.022				0.61				0.63
Low risk	53.9	3.19 (1.44-7.05)	0.004			7.4 (5.3-9.5)	1 (reference)			12.7 (9.6-15.8)	1 (reference)		
High risk	26.8	1 (reference)				2.2 (1.1-3.3)	2.36 (1.58-3.51)	<0.001		3.4 (1.4-5.5)	2.56 (1.69-3.88)	<0.001	
CEL				0.765 (0.68–0.84)	<0.001				0.69				0.73
Good	77.8	14.00 (5.12-38.28)	<0.001			14.3 (11.5-17.1)	1 (reference)	<0.001		18.9 (15.6-22.2)	1 (reference)	<0.001	
Intermediate	32.7	1.93 (0.72-5.15)	0.184			3.9 (2.0-5.7)	2.41 (1.54-3.75)	<0.001		7.4 (2.1-12.6)	2.70 (1.65-4.41)	<0.001	
Poor	20.0	1 (reference)	<0.001			2.2 (1.5-2.8)	3.96 (2.46-6.35)	<0.001		2.9 (1.0-4.7)	5.57 (3.29-9.43)	<0.001	

## Discussion

ICI therapy is considered a milestone in the history of NSCLC treatment and has achieved better therapeutic efficacy than traditional chemotherapy. However, only some patients benefit due to the lack of comprehensive biomarkers. ﻿Considering the high cost and the large number of patients with NSCLC, there is an urgent need to develop a risk-scoring system to identify candidates for ICI treatment. Thus, our study retrospectively investigated the factors associated with OS to establish and verify a novel risk-scoring system. After a broad analysis of 20 total clinical and laboratory parameters, the CEL score was developed and compared with validated scoring systems such as RMH, GRIm, mGPS, and LIPI. Our scoring index showed significant superiority over the other scoring systems in terms of discriminative and predictive ability. 

The RMH and GRIm scores were originally developed to support the selection of eligible patients enrolled in phase I trials of new treatment agents, and both were validated in patients with advanced NSCLC who were treated with ICIs [16,19]. ﻿The LIPI and mGPS are composite prognostic scoring indices that have been validated in patients with advanced NSCLC [7,11,12]. These indices consist of independent prognostic variables for ICI therapy and higher LDH levels, a decrease in albumin, a higher NLR, and more metastatic sites associated with shorter PFS and OS in patients with NSCLC. Serum albumin levels, NLR, and more metastatic sites showed no independent association with OS in our patient cohort. The CEL scoring index consists of indicators of inflammation and tumour burden. Inflammation promotes cancer progression and ICIs resistance [25-27]. CRP is an acute-phase protein of hepatic origin that reflects systemic inflammation. Several studies revealed that patients who had increased baseline CRP had worse survival than their normal counterparts [6,13]. LDH also reflects inflammation and tumour burden, particularly in patients with metastatic melanoma. Increased baseline LDH was associated with worse OS in patients with advanced NSCLC and was proposed as a risk factor for the LIPI score [6,11,12]. Poor ECOG PS is an indicator of greater disease burden and aggressive tumour biology. Randomised controlled trials excluded ECOG PS ³2 patients. However, routine everyday practice is quite different from randomised trials, and in our study, 56.8% of patients had poor ECOG PS. Poor ECOG PS was associated with adverse outcomes, consistent with our study results [9].

Our cohort included treatment-naive and platinum-pre-treated patients. The median PFS and OS were consistent with those of previously reported mixed cohort studies [28]. The CEL scoring index has a similar discriminative ability for platinum-refractory and treatment-naive patients. Therefore, we believe that the CEL index is convenient for both patient populations. In our study, the median OS was only 2.9 months for the poor-risk group. Although patients treated with conventional chemotherapy alone were not included in our study, the 2.9-month median OS raises questions regarding the contribution of ICIs. ﻿Therefore, we should consider omitting ICIs in this patient group. In our study, the good CEL risk group, which represented 36.7% of our cohort, was associated with a 77.8% ORR. To the best of our knowledge, this is the highest response rate reported in ICI studies. Currently, the PD-L1 level is the only available validated stratification biomarker in patients with NSCLC without driver mutations. The rate of high PD-L1 expression is ~30%, and ICIs with or without chemotherapy is associated with an ORR of 44.8%-60% [1,2,29]. Therefore, the CEL scoring index identified more patients with a significantly higher response rate than that of PD-L1.

In our study, 20.0% of patients had liver metastasis, which is similar to cornerstone ICI studies [1,30]. Although this variable showed no statistically significant association with OS in our study, it may be related to the relatively low patient volume. Liver metastasis is a well-known poor prognostic factor, and we think that it could be integrated into CEL scoring in studies to be conducted in cohorts with a larger number of patients [30].

This study has several limitations. First, this was a retrospective, bicentric study with a relatively small sample size. Second, although the CEL scoring index has a similar discriminative ability for treatment-naive and platinum-refractory patients, there was a heterogeneous profile in terms of patients and treatments in our cohort. Third, more than half of the patients’ PD-L1 levels in our study were not studied. If the PD-L1 level of all patients was available, a clearer conclusion could be obtained for the comparison between the CEL scoring index and PD-L1. Prospective, multi-institution external validation of the CEL scoring index is currently planned.

## Conclusions

The CEL score is a promising prognostic and predictive index consisting of serum CRP levels (C), ECOG PS (E), and serum LDH levels (L). This represents another step forward in the treatment of patients with advanced NSCLC. Further multi-institutional external validation studies are required.
